# An *in vitro* study of osteoblast vitality influenced by the vitamins C and E

**DOI:** 10.1186/1746-160X-8-25

**Published:** 2012-09-28

**Authors:** Kent Urban, Hans J Höhling, Beate Lüttenberg, Thomas Szuwart, Ulrich Plate

**Affiliations:** 1 Department of Cranio-Maxillofacial Surgery, University of Münster, Münster, Germany; 2 Institute of Medical Physics and Biophysics, University of Münster, Münster, Germany

**Keywords:** Bone cells, Osteoblast, Ascorbic acid, Tocopherol, Bone regeneration

## Abstract

Vitamin C and vitamin E are known as important cellular antioxidants and are involved in several other non-antioxidant processes. Generally vitamin C and vitamin E are not synthesized by humans and therefore have to be applied by nutrition. The absence or deficiency of the vitamins can lead to several dysfunctions and even diseases (e.g. scurvy). The main interest in this study is that vitamin C and E are known to influence bone formation, e.g. vitamin C plays the key role in the synthesis of collagen, the major component of the extracellular bone matrix.

In the present study we evaluate the effect of ascorbic acid (vitamin C) and α-tocopherol (vitamin E) on the proliferation and differentiation of primary bovine osteoblasts *in vitro*. Starting from standard growth medium we minimized the foetal calf serum to reduce their stimulatory effect on proliferation.

An improved growth and an increased synthesis of the extracellular matrix proteins collagen type I, osteonectin and osteocalcin was observed while increasing the ascorbic acid concentration up to 200 μg/ml. Furthermore the effects of α-tocopherol on cell growth and cell differentiation were examined, whereby neither improved growth nor increased synthesis of the extracellular matrix proteins collagen type I, osteonectin and osteocalcin were detected.

Further investigations are necessary to target at better supportive effect of vitamins on bone regeneration, and healing.

## Background

Diseases like osteoporosis will become more and more a major public health threat in the near future, e.g. in the demographic aging trend. The prevention and an effective treatment against osteoporosis and other bone-associated diseases is therefore one of the aims in the field of medical research. Another challenge is the *in vitro* formation of bone in the field of tissue engineering.

Proliferation and differentiation of osteoblasts enable the production of extracellular matrix (ECM) and is therefore the initial step to generate calcified tissue, especially bone. During the stages of differentiation, several proteins are synthesized by the osteoblasts, like collagen I, the main component of the ECM, and non-collagenous proteins like alkaline phosphatase, osteonectin and later in the differentiation progress osteocalcin. Up to now the scientific world focuses on the elucidation of the metabolic pathways during biomineralization to get an idea of how to promote the process of mineralization *in vivo* and *in vitro*. This could in the end lead to new applications in the coating of implants and prostheses or to an improvement in the treatment of bone diseases like osteoporosis. One qualified approach to reach this is to investigate the influence of different substances on the proliferation and differentiation of osteoblast like cell cultures *in vitro*. In this study, we examined two vitamins that cannot be synthesised by humans: ascorbic acid and vitamin E in form of α-tocopherol.

*In vivo*, ascorbate appears to be important as an antioxidant, and it is well-known that ascorbic acid acts as a cofactor for proline hydroxylase and lysine hydroxylase, enzymes involved in the process of collagen hydroxylation [1,2]. Ascorbic acid plays a role in reducing the iron prosthetic group of the hydroxylases and seems to be essential for the normal formation of bone. It was shown that the plasma membrane of osteoblasts possesses Na^+^ dependent transporter proteins specific for ascorbic acid by which the intracellular amount of ascorbic acid can be regulated [3]. *In vitro* studies show that supplementation of the medium with ascorbic acid stimulates procollagen hydroxylation, the processing and the fibril assembly [4] as well as the proliferation and differentiation of several types of cells [5-8]. The mechanism behind the influence of ascorbic acid on the proliferation and differentiation of various cell types still remains unclear. Besides the promoting effect of ascorbate, it was also shown that ascorbate was cytotoxic for cells, inducing for example apoptosis in HL-60 cells [9]. It was shown that the ECM, especially collagen I, increases the expression of differentiation markers in different cell types. This could be due to the presence of mature collagen alone, the amount of collagen-binding integrins or other pathways that could also be involved [10].

As second supplement we analysed the influence of vitamin E (α-tocopherol) on the proliferation and differentiation of our bovine cell culture. α-tocopherol is known for its antioxidant function through the reduction of free radicals, influencing the permeability of cell membranes, stabilizing cells in culture and is regarded to play a role in wound healing [11,12]. Beyond the antioxidant properties of α-tocopherol other effects due to specific interactions of this vitamin E with proteins like enzymes or transcription factors are possible [13]. It is also discussed that α-tocopherol somehow specifically enhances the effect of ascorbic acid on cells [14,15].

## Methods and materials

Most cell culture media, e.g. osteoblasts, are routinely supplemented with serum as a growth factor requirement. Therefore, we first tested our culture system regarding decreasing concentrations of fetal calf serum (FCS) (10% to 2%) by stopping proliferation of the cells. However, our results showed sufficient proliferation of osteoblasts in the 4% FCS.

For all experiments, medium S (standard) was used (High growth enhancement medium, GEM, without L-glutamine; MP Biomedicals, Eschwege, Germany). Medium S was supplemented with 4% FCS (Biochrom, Berlin, Germany) as explained above, 10.000 IU/ml penicillin, 10.000 μg/ml streptomycin, 250 μg/ml amphotericin B and 200 mM L-glutamine (Biochrom KG seromed, Berlin, Germany).

On the basis of medium S, 8.64 mg/ml β-glycerophosphate and 25 μg/ml ascorbic acid were added to achieve medium M. Ascorbic acid (vitamin C; Sigma-Aldrich, München, Germany) and vitamin E (Eastman Vitamin E-TPGS-NF Grade Eastman Chemical Workington UK) were added in different concentrations, respectively.

### Primary osteoblast like cell culture

Primary bovine osteoblast-like cells were used in this study. These cells were derived from the periosteum of calf metacarpus according to the instructions of [16]. Tissue explants were cultured for 4 weeks in medium S (High Growth Enhancement Medium, MP Biomedicals GmbH, Eschwege, Germany) supplemented with 10% FCS, 10.000 IU/ml penicillin, 10.000 μg/ml streptomycin, 250 μg/ml amphotericin B, 10 mM ß-glycerophosphate and 200 mM L-glutamine (Biochrom KG seromed, Berlin, Germany), at 37 °C and 5% CO_2_ in humidified air. The medium was replaced once a week. When the cells reached confluence, they were harvested (20 min incubation at 37 °C with 0.4 g collagenase, 98.8 mg HAM's F10 in 10 ml HEPES (2-[4-(2-hydroxyethyl)-1-piperazinyl]ethanesulfonic acid), repeated washing with phosphate-buffered saline (PBS), subsequently incubated for 15 min) and centrifuged. Pellets were resuspended in PBS and the cell number was determined in a coulter counter (CASY®I Modell TT, Schärfe System, Reutlingen, Germany).

### Cell proliferation

10.000/cm^2^ osteoblasts were seeded on 60 x 15 mm plastic petri dishes (Nunc, Roskide, Denmark) and cell proliferation was determined after 5 days, respectively. Cell morphology evaluation was performed by means of light microscopy. To determine the cell number digital photos were taken under standardized conditions and cells were counted using the software program Image J (Freeware) with the Plug-in Cell Counter.

#### Cell proliferation with vitamin C

The basis for these investigations was the cell-culture medium S supplemented with 4% FCS. To obtain medium M (mineralization) 8,64 g/L β-glycerophosphate and 0,1 g/L L-ascorbic acid were added to medium S. Different concentrations of ascorbic acid were added to the culture medium M to yield medium M with 25 μg/ml ascorbic acid, M1 with 100 μg/ml ascorbic acid, M2 with 200 μg/ml ascorbic acid, M3 with 300 μg/ml ascorbic acid, M4 with 400 μg/ml ascorbic acid, M5 with 500 μg/ml ascorbic acid.

#### Cell proliferation with Vitamin E

In this study and also by others d-alpha-tocopheryl polyethylene glycol 1000 succinate (TPGS) was used [17,18].

Typical properties of Vitamin E TPGS 1000: molecular weight, approx. 1,513; vitamin E content, mg/g, min. 260–300 as d-alpha-tocopherol; solubility in water miscible in all parts, a water-soluble form of vitamin E.

In these experiments medium M with and medium S without ascorbic acid were used, supplemented with 4% FCS respectively. Preliminary experiments showed that supplementation of the media with more than 20 μg/ml prevented any viability of the osteoblasts (Data not shown). For this reason vitamin E was used in lower concentration of 0,16 μg/ml (0,43 μmol/L TPGS). Such amount of TPGS was shown to be appropriate for cell culture also by others [17].

### Immunohistochemistry

To test the osteoblastic cell differentiation, collagen I, osteonectin and osteocalcin were assessed by immunohistochemistry. 60.000 osteoblasts/cm^2^ were seeded in 100 x 20 mm plastic petri dishes (TPP, Trasadingen, Schweiz). After cultivation for 14 days at 37 °C in an atmosphere of 5% CO_2_ in the different media, osteoblast-like cells were fixed with methanol and primary antibodies were used (diluted 1:100 with Blocking Solution): anti-collagen I (Biotrend, Cologne, Germany), anti-osteocalcin (TaKaRa Bio, MoBiTec, Gottingen, Germany), anti-osteonectin (TaKaRa Bio, MoBiTec, Goettingen, Germany). For the Dako-En-Vision-System, Dako Cytomation Envision + System Labelled Polymer (HRP) anti rabbit collagen 1 and HRP anti-mouse osteonectin and osteocalcin (Dako, Hamburg, Germany) were applied. Digital images were taken under standardized conditions by fluorescence microscopy (Axioplan 2 Carl Zeiss, Germany) and processed using the software Axio Vision 3.1 (Carl Zeiss, Germany).

## Results

### Cell proliferation test with fetal calf serum

**Figure 1:**
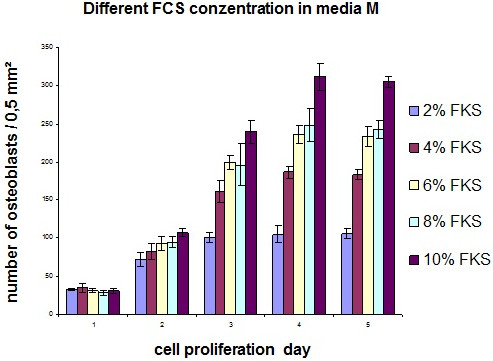
Influence of different FCS-concentrations (2% up to 10% FCS) on the cell proliferation of osteoblast like cells cultivated in media M over 5 days.

For this the medium M was used. Medium M was supplemented with increasing concentrations of fetal calf serum (FCS) from 2–10% to get information about its influence on cell vitality and cell proliferation. As shown in Figure 1 increase in cell proliferation correlated with the concentration of FCS in the medium (2%-10% FCS), however occurred even in medium with only 2% FCS.

**Figure 2:**
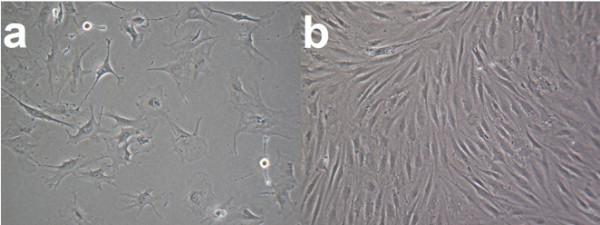
Cell morphology of osteoblast like cells (M x100) with 4% FCS (a = after 1 day and b = after 5 days in culture, medium M).

The highest proliferation of osteoblast like cells was determined in medium M with 10% FCS, indicated by the highest cell number detected after 2, 3, 4, and 5 days in culture (Figure 1). However, proliferation of the cells was already observed with 2% and 4% FCS (Figure 1 and Figure 2), respectively. To minimize the promoting effect of FCS on the proliferation of the osteoblast-like cells, all following experiments of this study were performed with medium M containing 4% FCS. On the one hand, this would guarantee proliferation of the osteoblast-like cells at all and, on the other hand, lead to apparent effects of the vitamins C and E on the cell proliferation in the further experiments.

As seen in Figure 2, the cell number of the osteoblast-like cells increases rapidly within 5 days in culture with medium M. Figure 2a shows single cells after one day with different shapes, widespread large cell bodies lying on the surface of the petri dishes. In Figure 2b cells after 5 days in culture show a sub-confluent cell monolayer with long cells lying next to each other.

### Cell proliferation with vitamin C

**Figure 3:**
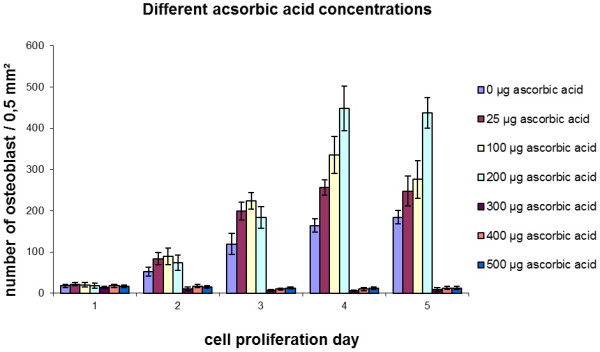
Cell proliferation of osteoblast like cells in medium with different ascorbic acid concentrations and medium S as reference (without vitamin C).

**Figure 4:**
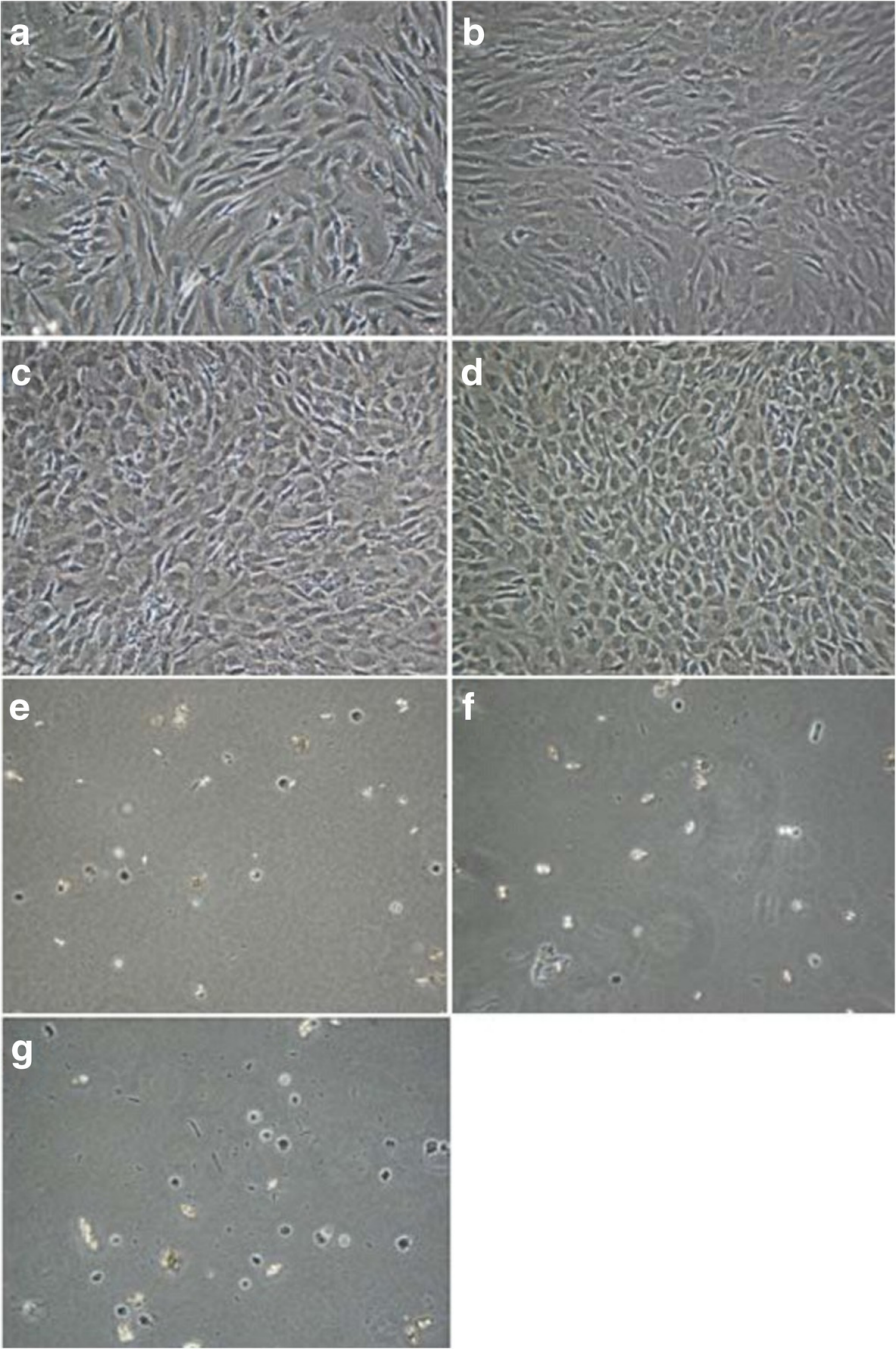
Osteoblast like cells (M x100) after 5 days in media with various ascorbic acid concentrations (a = 0.0 μg/ml, b = 25 μg/ml, c = 100 μg/ml, d = 200 μg/ml, e = 300 μg/ml, f = 400 μg/ml, g = 500 μg/ml).

Cell proliferation was assessed for the different concentrations of ascorbic acid. An increase in cell number was observed for all media with vitamin C addition from 0.0 μg/ml up to 200 μg/ml. (Figure 3). In culture media with ascorbic acid concentrations of more than 300 μg/ml nearly no cell proliferation was detected. This was confirmed by light microscopy (Figure 4). High amounts of osteoblasts were visible in the media with ascorbic acid additions up to 200 μg/ml (Figure 4a-d), respectively, whereas nearly no cells were seen in media with ascorbic acid additions from 300 μg/ml and more (Figure 4e-f).

Figure 4a and b show sub-confluent cell layers after 5 days in culture with no and 25 μg/ml ascorbic acid in the media. In Figure 4c and d the cells have reached confluence and show characteristic cell morphology with cuboidal appearance (100 μg/ml and 200 μg/ml), typical of osteoblast-like cells. Figure 4e, f and g show only a few cells with rounded shape (300 μg/ml and more).

### Immunohistochemical analysis

**Figure 5:**
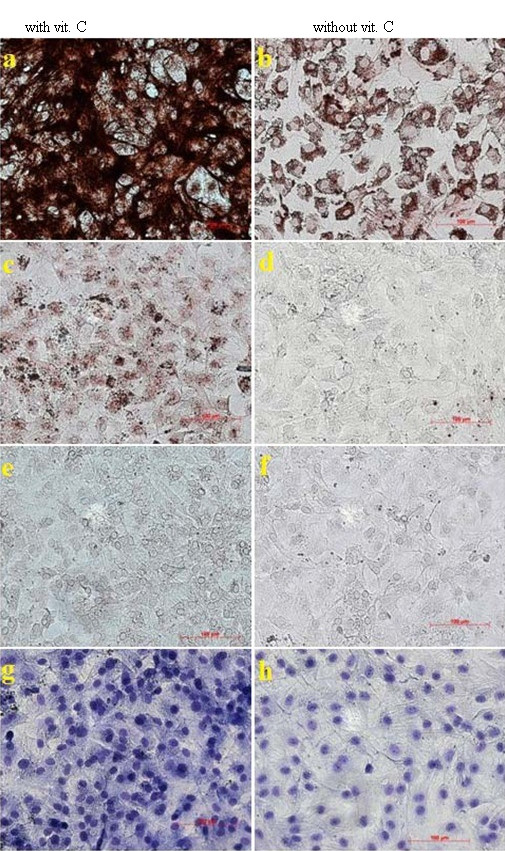
Immunohistochemical analyses (a-f) of collagen I (a, b), osteonectin (c, d), osteocalcin (e, f) of osteoblast like cells (staining toluidine blue) (g, h) in media with 200 μg/ml ascorbic acid (left column) and without ascorbic acid (right column) after 14 days.

After 14 days of cell culture, the three typical bone cell proteins collagen I, osteocalcin and osteonectin were determined by immunohistochemical investigations. With increasing ascorbic acid concentration of the medium also the amount of determined collagen I increased (Table 1 and Figure 5). Such clear correlation was not observed for osteocalcin and osteonectin. However as documented in Table 1 and Figure 5, an increase of osteonectin was detectable in media supplemented with high concentrations of ascorbic acid (200 μg/ml) compared to these proteins expressed in media S (without ascorbic acid). These results were confirmed by further immuno-histochemical analysis after 20 days (data not shown). The osteoblasts reached confluence in culture with 200 μg/ml ascorbic acid in the media (Figure 5a, c, e). Most of the cells showed a typical cuboidal shape. Figure 5b, d and f demonstrate sub-confluent cell layers (without ascorbic acid).

**Table 1:** Protein expression of osteoblast like cells in media S and media M (roughly estimated from immunhistochemical results (0 = no signal, 1 = weak signal, 2 = middle signal, 3 = strong signal))

	**Collagen 1**	**osteonectin**	**osteocalcin**
Media S without ascorbic acid	1	0	0
Media M with 25 μg/ml ascorbic acid	1	1	0
Media M1 with 100 μg/ml ascorbic acid	2	1	0
Media M2 with 200 μg/ml ascorbic acid	3	2	0

### Cell proliferation with vitamin E (α-tocopherol)

**Figure 6:**
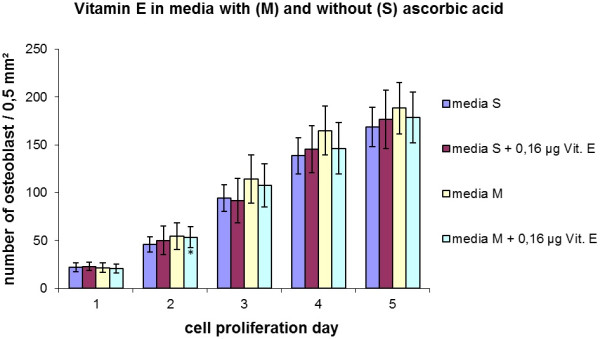
Cell proliferation of osteoblast like cells in media S and media M (S without and M with 25 μg/ml ascorbic acid) with and without 0,16 μg/ml vitamin E.

**Figure 7:**
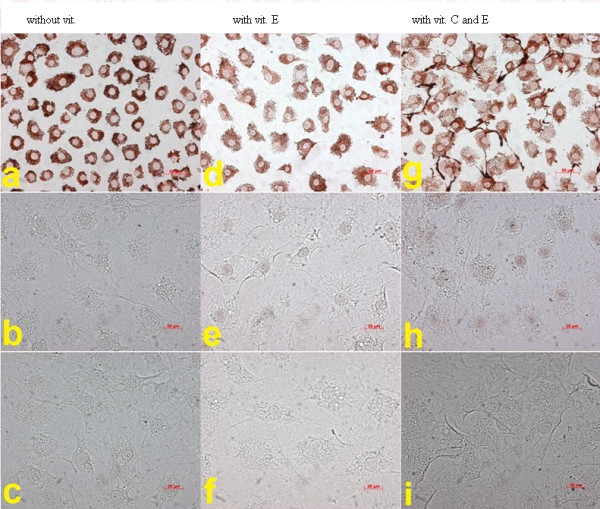
Immunohistochemical analyses of characteristic extracellular bone proteins: collagen I (a, d, g), osteonectin (b, e, h), osteocalcin (c, f, i) in media S without vitamin C and E (a-c), with 0,16 μg/ml vitamin E (d-f) and media M with 25 μg/ml vitamin C and 0,16 μg/ml vit. E (g-i) after 14 days.

An increase in cell proliferation due to the addition of α-tocopherol to the media was not observed in any of the experiments performed within this study. Other α-tocopherol concentrations in various preliminary tests also showed no increase in cell proliferation (data not shown). Figure 6 shows an increase in cell number over a period of 5 days with and without vitamin E and C. No effects concerning the composition of the media and cell proliferation were measurable. In addition, the results are in good correspondence with the data of Figure 3 at the same concentration of ascorbic acid (medium M = 25 μg/ml ascorbic acid). Table 2 and Figure 7 show the results after 14 days of cell culture with and without vitamin C and E. Collagen I, osteocalcin and osteonectin were determined immunohistochemically. Whereas the expression of collagen I was clearly visible under the used conditions, osteonectin and osteocalcin were not detectable. The proliferation of the osteoblasts reached sub-confluent cell layers.

**Table 2:** **Difference of protein expression between media S and media M of osteoblast like*cells (0 = no signal, 1 = weak signal, 2 = middle signal, 3 = strong signal)***

	**Collagen 1**	**osteonectin**	**osteocalcin**
Media S without Vit. C and without Vit. E	1	0	0
Media S without Vit. C and 0,16 μg Vit. E	1	0	0
Media M with 25 μg/ml Vit. C and 0,16 μg Vit. E	1	0	0

## Discussion

### Minimizing of fetal calf serum concentration

Fetal calf serum (FCS) is a highly complex composition of different nutrients and lots of other components, e.g. various enzymes or growth factors. Because FCS strongly stimulates cell proliferation, we reduced the concentration of FCS. The highest proliferation of osteoblast-like cells was observed in the media with 10% FCS as described in the literature [19,20]. Also, the typical appearance of cell morphology in media with 10% FCS of osteoblasts was shown. At FCS concentrations from 8% to 2% cell proliferation was observed, however the amount of cells decreased continuously. For our further experiments we chose 4% FCS. It was also shown that the morphology of the osteoblast-like cells at lower FCS concentrations does not change.

### Effect of ascorbic acid

In the experiments with vitamin C the promoting effect of ascorbic acid on the cell proliferation and also the differentiation of osteoblast-like cells were shown. A similar effect of vitamin C on osteoblasts was also described by others [7,21,22]. [21] investigated additions of 5 μg/ml, 10 μg/ml, 20 μg/ml, 50 μg/ml, and 100 μg/ml vitamin C to the cell culture medium, respectively. In their studies about the effect of vitamin C on human osteoblasts they found the highest cell proliferation at a vitamin C concentration of 50 μg/ml. The results of the present study showed that the osteoblasts proliferate with increased concentrations from 0 μg/ml - up to 200 μg /ml vitamin C concentrations; the highest proliferation rate was detected at a level of 200 μg/ml. Other basic media, a longer cultivation period, as well as different types of cells (human osteoblast-like instead of bovine osteoblast-like cells) in the study by [21] can be the reason for different results.

Furthermore, our *in vitro* experiments showed that vitamin C in concentrations of 300 μg/ml and higher acts “toxic” on bovine osteoblasts. Such cytotoxic phenomenon of ascorbate was also described by others [9,23], however the apoptosis-inhibiting effect of ascorbate was documented as well [24,25]. One study showed that not the ascorbate itself but the accompanying amount of generated H_2_O_2_, dependent on the media used, was responsible for the cytotoxic effect [26]. This could also explain the observed cell deaths in our study which can be due to high amounts of H_2_O_2_ coming from the high concentration of ascorbate in the media used. If there is too much H_2_O_2_, the enzyme catalase is not able to digest all of it into water and oxygen and this leads to cell death. From this it follows that the adverse effects of ascorbate seem to be the result of the chosen *in vitro* conditions and thus are no good hint to predict the effect of ascorbate in any situation *in vivo*. This process cannot occur *in vivo* because vitamin C excess will be excreted. In our *in vitro* investigations such excretion was impossible and this could be the reason for the observed “toxic” effect. Based on our results we can assume that bone cells grow slower in absence than in presence of vitamin C. Thus, vitamin C deficiency can inhibit bone formation on both levels, proliferation of the osteoblasts and differentiation to generate bone matrix and can be a possible candidate to favor osteoporosis.

The extracellular matrix proteins collagen type I, osteocalcin and osteonectin are decisive for bone formation and bone remodeling. Inhibition of bone formation and injury of connective tissue caused by a vitamin C deficiency is accompanied with a decreased collagen biosynthesis ([21], 27, [9]). Vitamin C plays an important role in hydroxylation of lysine and proline in the collagen biosynthesis, for example. In these reactions, which depend on vitamin C, α-ketoglutarate, O_2_ and Fe^2 +^ ions and the OH group will be attached to lysine and proline. The stability and strength in the connective tissue are dependent on these OH groups, because they are responsible for the binding of collagen and thus for the hydrogen bonds between the collagen polypeptide chains. For healing of wounds and bone fractures collagen is essential particularly. The special importance of vitamin C in collagen biosynthesis was confirmed in the investigations of several research groups [9,21,27].

[21] found that the highest synthesis of collagen type I, which is one of the main components of bone matrix, occur at a 50 μg/ml vitamin C concentration. In other media with 100 μg/ml vitamin C, they observed a lower rate of collagen biosynthesis. Our investigations confirmed the stimulation of the production of collagen. However, in our experiments we used vitamin C concentrations up to 200 μg/ml and therefore cannot confirm the highest collagen formation at 50 μg/ml vitamin C concentrations; the synthesis of extracellular matrix-relevant proteins in various concentrations of vitamin C should be analyzed in future studies. Also both matrix proteins osteocalcin and osteonectin were detected in cells with vitamin C. The mineralization process will be initiated by the formation of osteocalcin [28].

Thus one can assume that bone mineralization is at least accelerated by vitamin C [27]. It was found that osteoblasts tend to increase the expression of osteonectin and osteocalcin with an addition of vitamin C. It can be concluded that the supplementation of 200 μg/ml vitamin C has a positive effect on the proliferation and also cell differentiation of osteoblasts *in vitro*. A suitable concentration of vitamin C seems to be useful to improve wound healing and bone regeneration.

### Effect of α-tocopherol

In the experiments with α-tocopherol, the effect on the proliferation and differentiation of bovine osteoblasts was examined. Vitamin E (α-tocopherol) is said to have positive characteristics to bone regeneration [29]. In experiments in rats, protection against bone loss was achieved [30]. In previous studies other positive characteristics of vitamin E were shown, e.g. protection against oxidation and aging by a provision against free radicals, which reduces the growth of osteoblasts and their differentiation, or by maintenance of bone growth [30-32]. Furthermore, an increase in bone strength without change in bone density was achieved by vitamin E [29,33]. Also, a higher proliferation of osteoblast-like cells was expected. However, in our investigations we could not find an increasing cell proliferation by α-tocopherol. One reason could be that the vitamin E concentrations as vitamin E-TPGS were so high, that vitamin E-TPGS appeared toxic to the cells. An increase of the bone-characteristic extracellular matrix proteins collagen type I, osteocalcin and osteonectin was not shown either. All different media with vitamin E showed a less immuno-histochemical detection of proteins (data not shown). Again, this result reinforces the suspicion that the chosen *in vitro* concentrations of vitamin E had toxic effects on the cells comparable to vitamin C *in vitro*. For this reason, we also used reduced vitamin E-TPGS concentrations. However, the lowest concentration of vitamin E-TPGS used in this study in the human physiological area showed no increase in cell proliferation or synthesis of extracellular bone matrix proteins. Perhaps, further investigations with much lower concentrations should also follow.

Another reason for the non-occurring higher proliferation and differentiation under influence of vitamin E may be the choice of the added vitamin E-TPGS. Possibly, the use of a D-α-tocopherol-succinate or a DL-α-tocopherol-acetate would lead to different results. TPGS, on the other hand, is an extremely hydrophilic compound that proved to be a useful source of alpha-tocopherol in certain clinical situations [18]. Vitamin E-TPGS and its constituent PEG (polyethylene glycol) were classified as innocuous for humans and animals in certain concentrations [34]. Soeta et al. showed that the expression of osteocalcin was decreased by treatment of alpha-tocopherol that was conjugated to bovine serum albumin [35]. These results indicate that vitamin E inhibits differentiation of osteoblasts especially from early stage to osteoid-producing stage.

Possibly, an improved cell growth and a longer lifetime of cells can be revealed by vitamin E with the awarded antioxidant activity.

## Conclusions

This work demonstrated a positive effect of vitamin C on the proliferation of primary bovine osteoblasts *in vitro*. Vitamin C also increased the synthesis of collagen I. A possible application of the analyzed vitamins could be the coating of implants. The bio-active vitamins from the implant surface can be transmitted directly into the wound. It can be supposed that this increased cell growth improves wound healing and bone regeneration. To increase proliferation and collagen formation, the culture of osteoblast like cells in vitamin-culture media can be confirmed as quite reasonable.

Our analyses also showed that vitamin E does not stimulate proliferation of osteoblasts or their production of collagen type I, osteocalcin and osteonectin.

Further investigations should follow with the aim to increase the supportive effect of vitamins on biological processes, such as wound healing, bone regeneration, and revised healing of bone implants.

## Competing interests

The authors declare that they have no competing interests.

## Authors` contributions

UP BL and TS have conceived and designed the study. HJH participated in the design. KU has prepared the cell cultures and measured the osteoblast vitality. UP has managed the study. All authors have contributed to the coordination of the study and helped to draft the manuscript. All authors read and approved the final manuscript.

## Authors' information

Kent Urban: Albert-Schweitzer-Campus, Gebäude: W30, 48149 Münster, Germany.

Hans Jürgen Höhling: Albert-Schweitzer-Campus, Gebäude: W30, 48149 Münster, Germany.

Beate Lüttenberg: Albert-Schweitzer-Campus, Gebäude: W30 48149 Münster, Germany.

Thomas Szuwart: Albert-Schweitzer-Campus, Gebäude: W30 48149 Münster, Germany.

Ulrich Plate: Albert-Schweitzer-Campus 1, Gebäude: W30, 48149 Münster, Germany Phone 0049251/8347122.
